# Addiction to Gambling and Antidepressants: Impact on Impulsivity and Compulsive Behavior

**DOI:** 10.1192/j.eurpsy.2026.10835

**Published:** 2026-07-31

**Authors:** P. S. Pires, C. Cunha, R. Cabral, F. Cunha, I. Santos, C. Bastos, I. Silas, S. Torres, A. P. Costa

**Affiliations:** Psychiatry, ULS Viseu Dão-Lafões, Viseu, Portugal

## Abstract

**Introduction:**

Pathological gambling disorder (PGD) is a psychiatric condition characterized by a loss of control over betting behavior, associated with dysfunctions in the dopaminergic and serotonergic systems. Antidepressants, particularly selective serotonin reuptake inhibitors (SSRIs) and serotonin-norepinephrine reuptake inhibitors (SNRIs), are frequently used to treat psychiatric comorbidities in these patients. However, their effects on impulsivity and compulsive behavior remain controversial. While some studies suggest a beneficial effect in reducing compulsivity, others indicate a possible exacerbation of impulsivity, potentially increasing the risk of pathological gambling.

**Objectives:**

To conduct a narrative literature review on the impact of antidepressants on pathological gambling behavior, discussing possible neurobiological mechanisms involved and implications for clinical management.

**Methods:**

A narrative search was conducted in databases such as PubMed, Scopus, and Web of Science, including studies published between 2015 and 2024. The review analyzed research addressing the impact of SSRIs, SNRIs, and dopaminergic antidepressants on PGD, as well as their effects on impulsivity regulation and inhibitory control.

**Results:**

Evidence suggests that SSRIs, such as fluoxetine and sertraline, may reduce compulsivity by modulating serotonergic neurotransmission and its interaction with the prefrontal cortex. However, their impact on impulsivity is variable, possibly due to the complex interaction between the serotonergic and dopaminergic systems within the reward circuitry. SNRIs and dopaminergic antidepressants, such as bupropion, have been associated with increased impulsivity in some studies, potentially exacerbating risk-taking behavior. The presence of psychiatric comorbidities, such as major depressive disorder and attention-deficit/hyperactivity disorder (ADHD), may influence treatment response, highlighting the need for a personalized approach.

**Conclusions:**

The effect of antidepressants on pathological gambling is not yet fully understood and may vary depending on the patient’s neurobiological profile and the class of medication used. Clinical management should consider individual differences and the presence of comorbidities, avoiding pharmacological strategies that may increase impulsivity. Future studies should further explore the underlying mechanisms of this relationship and guide more effective therapeutic approaches.

**Disclosure of Interest:**

None Declared

